# Anethole Attenuates Enterotoxigenic *Escherichia coli-*Induced Intestinal Barrier Disruption and Intestinal Inflammation via Modification of TLR Signaling and Intestinal Microbiota

**DOI:** 10.3389/fmicb.2021.647242

**Published:** 2021-03-25

**Authors:** Qingyuan Yi, Jiaxin Liu, Yufeng Zhang, Hanzhen Qiao, Fang Chen, Shihai Zhang, Wutai Guan

**Affiliations:** ^1^Guangdong Province Key Laboratory of Animal Nutrition Control, College of Animal Science, South China Agricultural University, Guangzhou, China; ^2^College of Animal Science and National Engineering Research Center for Breeding Swine Industry, South China Agricultural University, Guangzhou, China; ^3^College of Biological Engineering, Henan University of Technology, Zhengzhou, China

**Keywords:** anethole, piglet, *Escherichia coli* K88, antibiotics, microbiota

## Abstract

This study aimed to investigate the effects of dietary anethole supplementation on the growth performance, intestinal barrier function, inflammatory response, and intestinal microbiota of piglets challenged with enterotoxigenic *Escherichia coli* K88. Thirty-six weaned piglets (24 ± 1 days old) were randomly allocated into four treatment groups: (1) sham challenge (CON); (2) *Escherichia coli* K88 challenge (ETEC); (3) *Escherichia coli* K88 challenge + antibiotics (ATB); and (4) *Escherichia coli* K88 challenge + anethole (AN). On day 12, the piglets in the ETEC, ATB, and AN group were challenged with 10 mL *E. coli* K88 (5 × 10^9^ CFU/mL), whereas the piglets in the CON group were orally injected with 10 mL nutrient broth. On day 19, all the piglets were euthanized for sample collection. The results showed that the feed conversion ratio (FCR) was increased in the *Escherichia coli* K88-challenged piglets, which was reversed by the administration of antibiotics or anethole (*P* < 0.05). The duodenum and jejunum of the piglets in ETEC group exhibited greater villous atrophy and intestinal morphology disruption than those of the piglets in CON, ATB, and AN groups (*P* < 0.05). Administration of anethole protected intestinal barrier function and upregulated mucosal layer (mRNA expression of mucin-1 in the jejunum) and tight junction proteins (protein abundance of ZO-1 and Claudin-1 in the ileum) of the piglets challenged with *Escherichia coli* K88 (*P* < 0.05). In addition, administration of antibiotics or anethole numerically reduced the plasma concentrations of IL-1β and TNF-α (*P* < 0.1) and decreased the mRNA expression of TLR5, TLR9, MyD88, IL-1β, TNF-α, IL-6, and IL-10 in the jejunum of the piglets after challenge with *Escherichia coli* K88 (*P* < 0.05). Dietary anethole supplementation enriched the abundance of beneficial flora in the intestines of the piglets. In summary, anethole can improve the growth performance of weaned piglets infected by ETEC through attenuating intestinal barrier disruption and intestinal inflammation.

## Introduction

Enterotoxigenic *Escherichia coli* (ETEC) is considered one of the main causes of diarrhea in weaning piglets (Fairbrother et al., 2005). Generally, a poor breeding environment causes an increase in intestinal ETEC (Yokoyama et al., 1992), disrupts the balance of intestinal flora (Li et al., 2012) and affects the digestion and absorption of nutrients (Gao et al., 2013). The enterotoxins secreted by ETEC can destroy the intestinal mucosa layer and tight junction structure, which leads to increased permeability of the intestine (Fleckenstein et al., 2010; Dubreuil, 2012). Bacteria or antigens that pass through the intestinal mucosa are captured and recognized by immune cells, which further activate the immune response and inflammatory process (Moretó and Pérez-Bosque, 2009; Turner, 2009). Damage to the intestine could reduce growth performance, cause severe diarrhea, and even lead to piglet death (Fleckenstein et al., 2010).

A large number of antibiotics are used in animal production worldwide each year, of which most are antibiotic growth promoters (AGPs). Based on a survey of antibiotic usage in China for 2013, total antibiotic production usage was approximately 162,000 tons, of which 52% was used in animals (Ying et al., 2017). According to the requirements of Chinese government, feed manufacturers is not allowed to produce commercial feeds containing growth-promoting drug feed additives since July 1, 2020.

”Medicine and food are homologous” comes from a view of traditional Chinese medicine, meaning that some of food can have a certain therapeutic effect. Anethole (AN) was originally extracted from fennel and has long been proven to have anti-inflammatory effects, and that it’s also has been used in animal production (Windisch et al., 2008). Previous studies have reported that AN can improve the growth performance of animals at an appropriate dosage (Kim et al., 2013; Zeng et al., 2015; Charal et al., 2016). However, to the best of our knowledge, there is no comprehensive report on the effects of AN on ETEC-infected piglets. Thus, the primary aim of this study was to determine the effects of AN on the growth performance, intestinal barrier function, inflammatory response and intestinal microbiota of piglets challenged with ETEC.

## Materials and Methods

### Ethics Approval

All the experimental protocols in this study were satisfy the needs of animal welfare and conducted in strict accordance with the Guidelines for the Protection and Use of Laboratory Animals issued by the South China Agricultural University Animal Care and Use Committee (No. 20110107-1, Guangzhou, China).

### Animals, Housing, and Experimental Design

This trial is conducted in an experimental house with a controlled temperature at 30 ± 2°C and humidity below 80%. Piglets were individually fed in metabolic cages (1.2 m × 0.4 m × 0.5 m) with a three-day adaptation period to adapt to the new environment and feed. All piglets had free access to feed and water. During the adaptation period, piglets did not show any symptoms of diarrhea, skin lesions and obvious inflammation, which indicated that piglets were healthy and suitable for this experiment. After adaption, 36 male piglets (Duroc × (Landrace × Yorkshire), initial weight 7.5 ± 1 kg) were assigned to one of four treatments according to the principle of similar weight (*n* = 9). This experiment last for 19 days. Four treatments are listed as follows: (1) sham challenge (CON); (2) *Escherichia coli* K88 challenge (ETEC); (3) *Escherichia coli* K88 challenge + antibiotics (ATB); and (4) *Escherichia coli* K88 challenge + anethole (AN). CON and ETEC groups receives the control diet, ATB group receives the control diet supplemented with antibiotics (50 mg/kg quinocetone, 75 mg/kg chlortetracycline, 50 mg/kg kitasamycin), and AN group receives the control diet supplemented with AN (300 mg/kg, pure AN ≧ 7.5%, coating with corn starch, Pancosma, Switzerland). The feed formula was prepared according to NRC (2012). The ingredient composition and nutrient levels of control diet are presented in Supplementary Table 1.

### Enterotoxigenic *Escherichia coli* K88 Challenge

*Escherichia coli* K88 (CVCC225) was purchased from the Chinese Veterinary Medicine Collection Center, and it was confirmed to have heat labile enterotoxin (LT), heat stable enterotoxin (ST), and F4 fimbriae in our laboratory (Ren et al., 2019). On day 12, piglets from the ETEC, ATB, and AN group orally challenged with 10 mL nutrient broth (NB) containing 5 × 10^9^ CFU/mL ETEC K88 via a syringe, CON group orally injected with 10 mL of sterilized NB. CON group was kept in isolation in order to avoid cross-contamination.

### Sample Collection

Blood and feces samples were collected on day 19. Five milliliter of blood samples were collected into tubes containing EDTA via the anterior vena cava puncture and quickly centrifuged (1000 × g, 4°C, 10 min) for plasma samples in 30 min, then stored at −80°C. At the same time, over 5 g fresh feces samples were collected into centrifuge tubes and stored at −80°C. After blood and feces samples collection, piglets were immediately euthanized. About 2 cm length of duodenum (about 10 cm near the pylorus) and jejunum (about 60 cm near the pylorus) were collected, then stored into 4% paraformaldehyde solution for histological analyses. Jejunal and ileal segments (10 cm length) were opened longitudinally and the contents were flushed in cold normal saline (NS) solution for twice. Mucosa was collected by scraping using a sterile glass microscope slide at 4°C, rapidly frozen in liquid N_2_ and stored at −80°C for the analysis of mRNA and protein expression. Similarly, mesenteric lymph node (MLN) was collected and rapidly frozen in liquid N_2_ for the analysis of mRNA expression. The time from anesthesia to complete sampling was controlled at about 30 min per piglet.

### Measurements

#### Growth Performance

Feed intake of each piglets was daily recorded. Body weight of each piglets were recorded on day 0, day 12, and day 19 to calculate average daily gain (ADG), average daily feed intake (ADFI) and F/G (Feed conversion ratio) respectively.

#### Immunological Parameters

Plasma IL-1β, TNF-α, IL-6, and IL-10 were analyzed by using commercially available porcine ELISA kits (Huamei, Wuhan), according to the manufacturer’s instructions. All assays were run in duplicate and diluted if necessary.

#### Intestinal Morphology

The samples of duodenum and jejunum were embedded in paraffin. Each sample was used to prepare one slide with two sections (4 μm thickness), which were stained with Hematoxylin-Eosin. Three views of each section and three well-oriented villi and crypts of each view were selected for intestinal morphology measurement. Villi height and crypt depth ratio (VCR) was calculated after measure.

#### Quantitative PCR for Gene Expressions

Total RNA was extracted from the frozen jejunum, ileum, and MLN tissues by using total RNA extraction kit (LS040, Promega, Shanghai, China) according to the manufacturer’s instruction. The quality, purity and concentrations of RNA samples were assessed by electrophoresis on 1.5% agarose gel (130 V, 18 min) and NanoDrop Spectrophotometer (A260/A280). Then, the RNA was adjusted to a uniform concentration by using RNase-free ddH_2_O. Subsequently, reverse transcription of the RNA to complementary cDNA was performed using a cDNA reverse transcription kit (RR047A, Takara, Tianjin, China). Quantitative PCR by using the SYBR green system (RR820A, Takara) was performed on QuantStudioTM 6 Flex (Applied Biosystems, CA, United States). The reaction mixture (10 μL) contained 5 μL of SYBR Green PCR Master Mix, 1 μL of cDNA, 0.4 μL of forward and reverse primer (10 μM/L), 0.2 μL of ROX Reference Dye II (50×), and 3 μL of RNase-free ddH_2_O. The PCR reaction was repeated three times for each gene and carried out as following: one cycle at 50°C for 120 s and 95°C for 600 s, forty cycles at 95°C for 15 s and 60°C for 60 s and one cycle at 95°C for 15 s, 60°C for 60 s, and 95°C for 15 s. Target gene expression was calculated based on the 2^–ΔΔ*Ct*^ method (Livak and Schmittgen, 2001) and normalized to GAPDH. The primer sequences were designed by using Primer 3.0 (Supplementary Table 2).

#### Western Blot Analysis for Protein Expressions

The total protein in the frozen jejunal and ileal tissue samples was lysed in RIPA (P0013B, Biyuntian, Shanghai, China). The protein concentration of each sample was measured using BCA protein assays (P0010, Biyuntian, Shanghai, China). Equal amounts of denatured protein (25 μg) from each sample were separated on 10% SDS-PAGE and then electroblotted onto PVDF membranes. Membranes were blocked for 2 h with 5% skimmed milk in TBST at room temperature. Subsequently, the membranes were incubated with specific antibody [ZO-1 (ab96b87, Abcam, United States), Occludin (ab31721, Abcam, United States), Claudin-1 (ab15098, Abcam, United States), and β-actin (bs-0061R, Bioss, China)] for 12 h at 4°C, and then incubate with secondary antibody for 1 h at room temperature. Finally, the proteins were detected using ECL chemiluminescence reagents (P1020, ApplyGen, Beijing, China) and FluorChem M Fluorescent Imaging System (ProteinSimple, CA, United States). Protein expression were analyzed by using image J software.

#### 16S rDNA Sequencing

Gut microbiome of feces was analyzed by High-throughput 16S rDNA sequencing technology. Sequencing (PE250, NovaSeq6000, Illumina Inc., CA, United States) was performed by Novogene Co., Ltd. (Beijing, China), the V3–V4 region of the 16S rDNA was amplified using primers 341F (5′-CCTAYGGGRBGCASCAG) and 806R (5′-GGACTACNNGGGTATCTAAT). For 16S rDNA sequencing data, statistical analyses were performed with NovoMagic (Novogene Co., Ltd.) online tools.

### Statistical Analysis

All data of this experiment were analyzed by using one-way ANOVA according to the general linear models (GLM) procedure of SPSS 22.0 (IBM Inc., United States). Data were expressed as means ± SEM. Comparisons between the values of mean treatment were made by LSD using Duncan’s multiple test. *P* < 0.05 was considered as statistically significant differences and as tendency when 0.05 < *P* < 0.10.

## Results

### Performance

After ETEC challenge, the piglets fed diets containing antibiotics or AN had lower (*P* < 0.05) F/G than the piglets challenged with ETEC, and this F/G was similar to that of the piglets given the CON treatment (Table 1).

**TABLE 1:** Effects of anethole on growth performance of piglets challenged with enterotoxigenic *Escherichia coli* K88.

Items	Treatments				SEM	*P-v*alue
	
	CON	ETEC	ATB	AN		
Initial BW (kg)	7.17	7.13	7.32	7.25	0.16	0.977
Final BW (kg)	13.78	12.69	13.60	13.51	0.41	0.795
Pre-challenge (0–12 days)						
ADFI (g)	357	358	355	371	18	0.990
ADG (g)	275	272	280	285	14	0.989
F/G	1.33	1.30	1.31	1.33	0.04	0.995
Post-challenge (13–19 days)						
ADFI (g)	623	559	623	608	27	0.832
ADG (g)	445	307	406	394	22	0.136
F/G	1.47^*b*^	1.87^*a*^	1.52^*b*^	1.55^*b*^	0.06	0.050

**n* = 9. Different letters mean statistically significant difference among the groups (*P* < 0.05).*

### Intestinal Morphology

**FIGURE 1:**
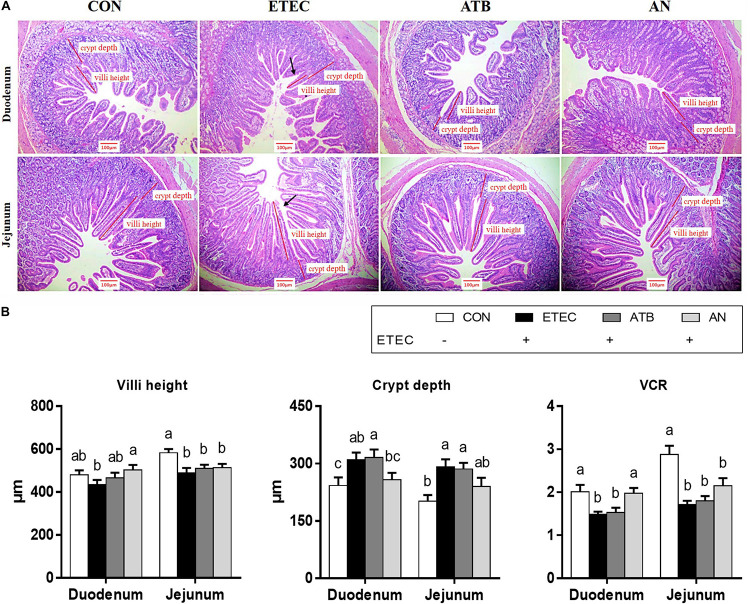
Effects of anethole on intestinal morphology of piglets challenged with enterotoxigenic *Escherichia coli* K88. **(A)** HE staining of intestine **(B)** Villi height, crypt depth and VCR of intestine. The data in each group is expressed as the mean ± SE (*n* = 9). Different letters mean statistically significant difference among the groups (*P* < 0.05).

The duodenums of the piglets in the AN group exhibited higher villus heights (*P* < 0.05) and VCRs (*P* < 0.05) than those of the piglets in the ETEC group. The piglets in the ATB group had greater duodenal crypt depths (*P* < 0.05) than those in the CON and AN groups. The jejunums of the piglets in the CON group had higher villus heights (*P* < 0.05) and VCRs (Figure 1, *P* < 0.05) than those of the piglets in the other groups. In addition, the piglets in the CON group had lower crypt depths (Figure 1, *P* < 0.05) than those in the ETEC and ATB groups.

### Barrier Function

**FIGURE 2:**
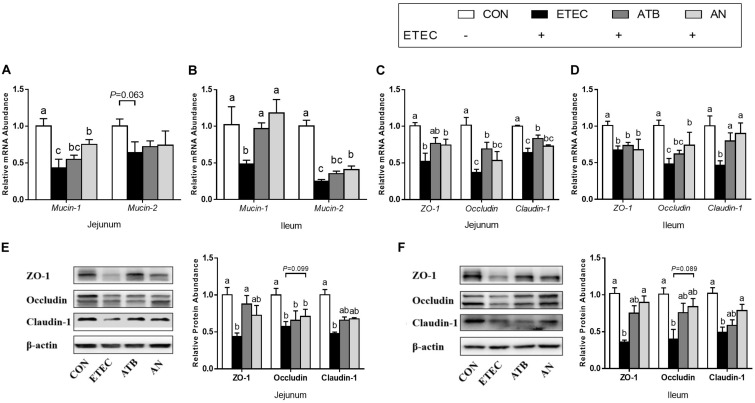
Effects of anethole on **(A–B)** intestinal secretory and **(C–F)** barrier function of piglets challenged with enterotoxigenic *Escherichia coli* K88. For PCR assays, *n* = 8, *GAPDH* as reference gene; For western blot, *n* = 3, β-actin as reference protein; The data in each group is expressed as the mean ± SE. Different letters mean statistically significant difference among the groups (*P* < 0.05).

The relative mRNA expression of mucin-1, mucin-2, ZO-1, Occludin, and Claudin-1 in the jejunum and ileum of the piglets was significantly downregulated (*P* < 0.05, Figure 2) in response to ETEC challenge (*P*_mucin–2_ = 0.063). However, this downregulation was partially mitigated by dietary supplementation with antibiotics or AN. In addition, the ZO-1, Occludin, and Claudin-1 protein levels were also markedly decreased (*P* < 0.05, Figure 2) after ETEC challenge, which can be attenuated by dietary supplementation with antibiotics or AN.

### Inflammatory and Immunological Responses

**FIGURE 3:**
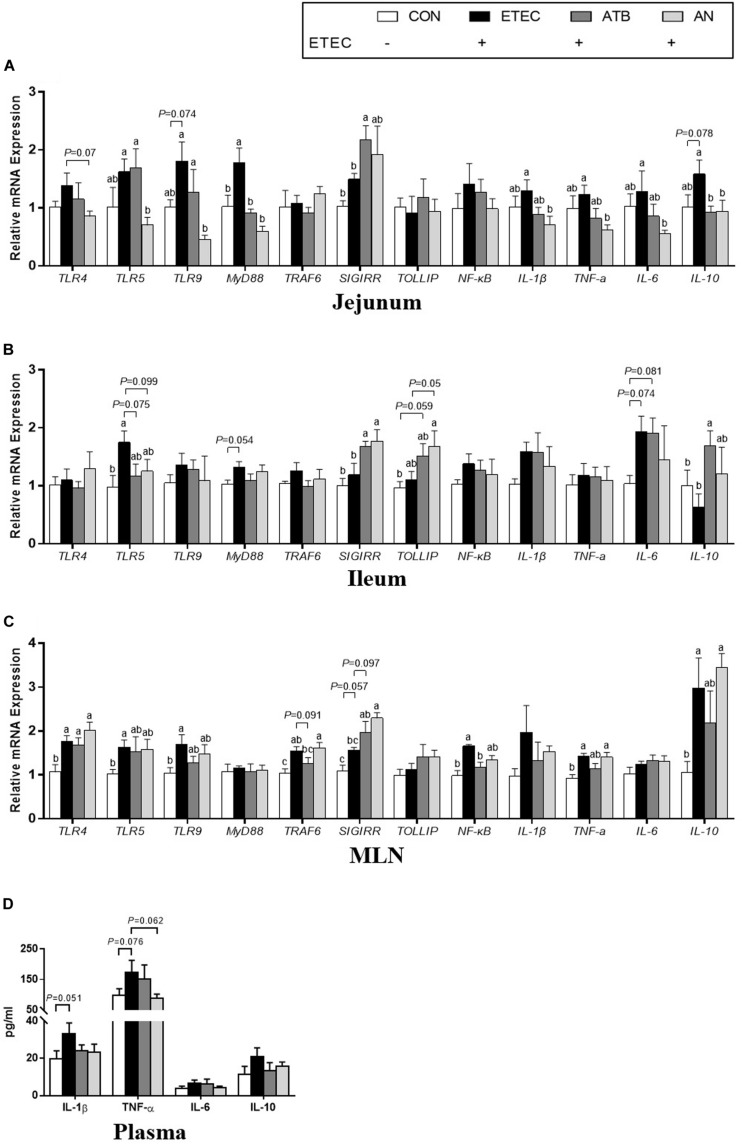
Effects of anethole on concentration of relative mRNA expression of genes related to inflammation **(A)** jejunum, **(B)** ileum, **(C)** MLN, and **(D)** plasma cytokine and of piglets challenged with enterotoxigenic *Escherichia coli* K88. ELISA for plasma, *n* = 9; For PCR assays, *n* = 8, *GAPDH* as reference gene; The data in each group is expressed as the mean ± SE. Different letters mean statistically significant difference among the groups (*P* < 0.05).

The plasma levels of IL-1β and TNF-α in the piglets tended to increase after ETEC challenge (*P* < 0.1), whereas this tendency was not observed in the ETEC-challenged piglets in the ATB and AN groups. Compared with those of the piglets in the CON group, the relative mRNA expression levels of certain genes were upregulated (*P* < 0.05) in the jejunum (TLR9, MyD88 and IL-10), ileum (TLR5), and MLN (NF-κB) of the piglets in the ETEC group, which could be attenuated by supplementation with antibiotics and AN. Compared with those of the piglets in the ATB group, the relative mRNA expression levels of certain genes was significantly decreased (*P* < 0.05) in the jejunum (TLR5 and TLR9) and significantly increased (*P* < 0.05) in the MLN (TRAF6) of the piglets in the AN group. In addition, compared with those of the piglets in the CON or ETEC group, the relative mRNA expression levels of genes related to the MyD88/NF-κB signaling pathway were upregulated (*P* < 0.05) in the jejunum (SIGIRR) and ileum (SIGIRR and IL-10) of the piglets in the ETEC group and the ileum (SIGIRR) and MLN (SIGIRR) of the piglets in the AN group (Figure 3).

### Gut Microbiome

**FIGURE 4:**
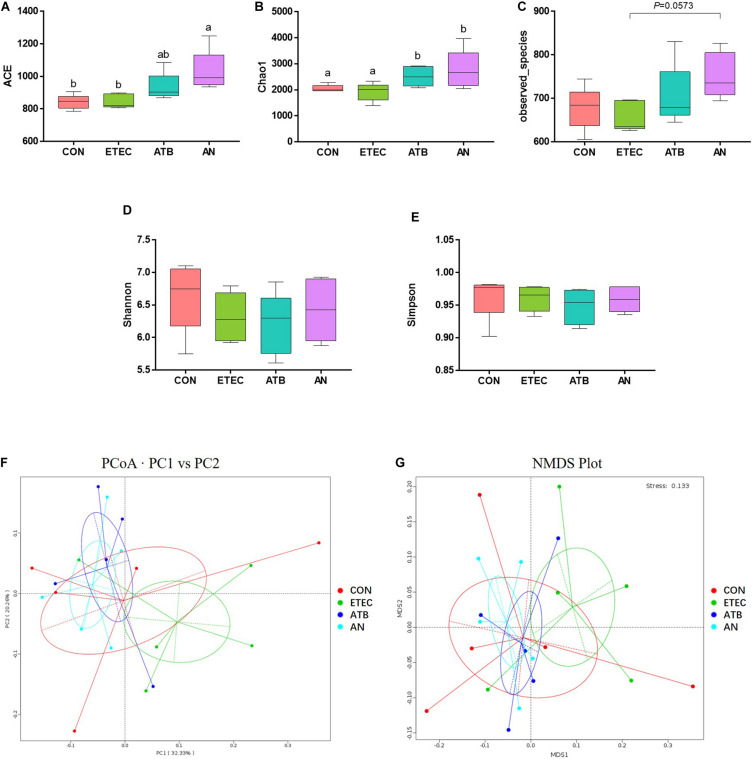
Effects of anethole on alpha diversity and beta diversity of fecal microbiota of piglets challenged with enterotoxigenic *Escherichia coli* K88 (*n* = 5). **(A–E)** Alpha Diversity index: ACE, Chao1, Observed_species, Shannon, Simpson. **(F)** Principal Coordinates Analysis based weighted Unifrac. **(G)** NMDS Plot Analysis based weighted Unifrac.

A total of 1,775,153 high-quality sequences were generated from 20 fecal samples (four treatments, *n* = 5), with an average of 88,758 sequences per sample, and 64,854 ± 2,566 effective tags were obtained for subsequent analysis after the noise sequences were discarded. Finally, all the effective tags were clustered to operational taxonomic units (OTUs) at 97% sequence similarity and then allotted to 23 phyla, 39 classes, 81 orders, 145 families, 322 genera, and 1,738 OTUs. For alpha-diversity, the bacterial richness ACE and Chao1 index of the AN group were markedly higher than those of the CON and ETEC groups (*P* < 0.05), the richness Observed_species index of the ETEC and AN group had a tendency of significant difference (0.05 < *P* < 0.10), the diversity Shannon and the Simpson index had no significant difference among four treatment groups (*P* > 0.05). For beta diversity, the PCoA (PC1 32.33% vs PC2 20.26%) and NMDS (stress = 0.133) analyses based on Weighted UniFrac distances showed that the microbiota from the piglets in the ETEC group obviously tended to separate from that of the piglets in both the ATB and AN group (Figure 4).

**FIGURE 5:**
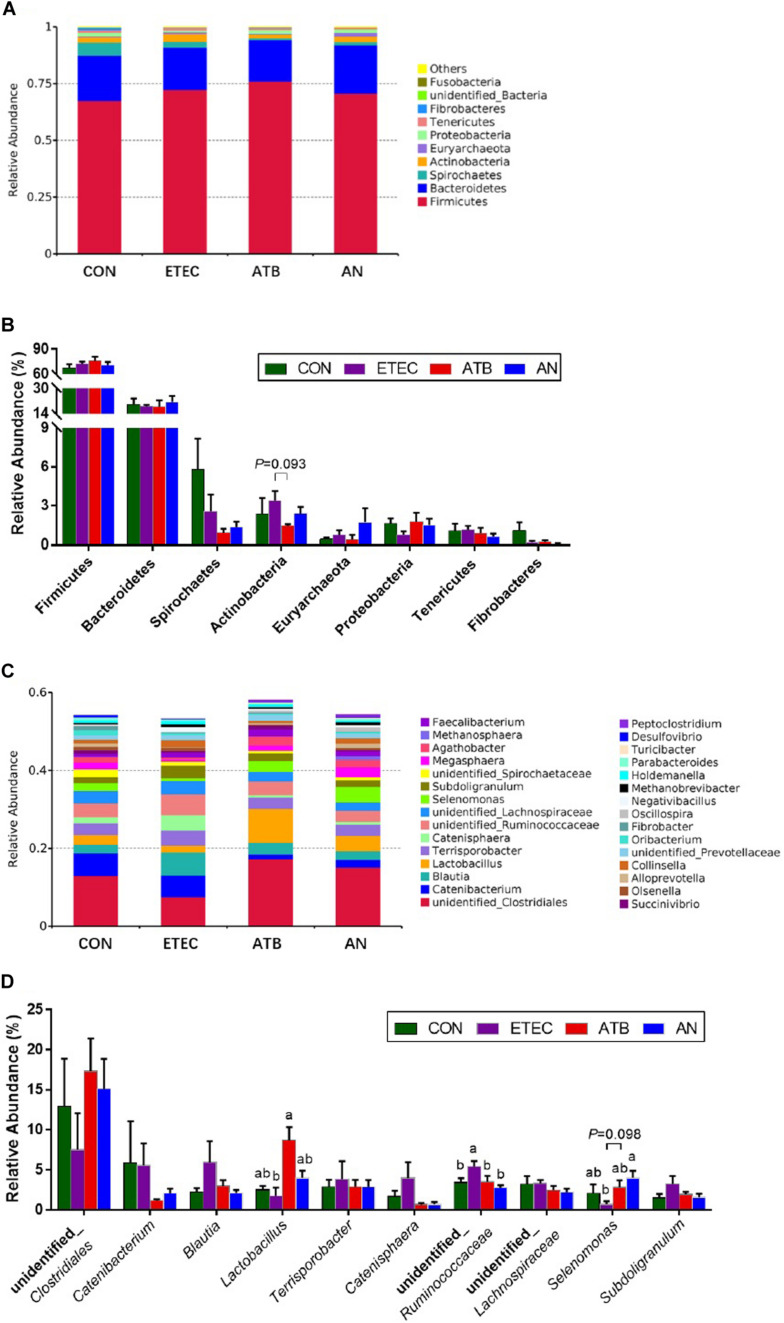
Effect of anethole on microbial composition of fecal microbiota of piglets challenged with enterotoxigenic *Escherichia coli* K88 (n = 5). **(A)** Relative abundance in phyla. **(B)** Top eight in phyla. **(C)** Relative abundance in genus. **(D)** Top 10 in genus.

At the phylum level, five major bacteria in the feces of piglets were Firmicutes (51.74–9.85%), Bacteroidetes (6.97–34.30%), Spirochaetes (0.35–13.06%), Actinobacteria (0.84–7.26%), and Euryarchaeota (0.01–5.69%). At the genus level, unidentified_*Clostridiales* (1.59–35.52%), *Catenibacterium* (0.30–16.21%), *Blautia* (0.81–15.16%), *Lactobacillus* (0.51–13.55%), *Terrisporobacter* (0.37–12.64%), and *Catenisphaera* (0.20–10.97%) were the most predominant genera in all the samples, and three genera (*Lactobacillus*, unidentified_*Ruminococcaceae*, and *Selenomonas*) were significantly different among the different groups on top 10 (Figure 5 and Supplementary Table 4). *Lactobacillus* abundance in the ATB group was significantly higher (*P* < 0.05) than that in the ETEC group. unidentified_*Ruminococcaceae* abundance in the ATB and AN groups was significantly lower (*P* < 0.05) than that in the ETEC group. *Selenomonas* abundance in the AN group was significantly higher (*P* < 0.05) than that in the ETEC group.

## Discussion

Enterotoxigenic *Escherichia coli* regulates the secretion of enterotoxins and induces diarrhea and intestinal impairment in weaned piglets (Che et al., 2017). In the present study, the FCR of piglets was significantly increased after ETEC challenge. Correspondingly, after statistical analysis of the internal morphology of piglets, we can find that after ETEC challenge, the VCR values of duodenum and jejunum of piglets in ETEC group were significantly reduced (*P* < 0.05), while the VCR value of duodenum of piglets in AN group did not decrease significantly, which is the most direct evidence that AN can help piglets resist ETEC infection. Maruzzella and Freundlich found that AN has the strong bacteriostatic effect (Maruzzella and Freundlich, 2010). Meanwhile, some other studies have found that AN inhibits the secretion of acetylcholinesterase (AchE) and increases the concentration of acetylcholine (Ach; Bhadra et al., 2011), high Ach levels trigger intestinal smooth muscle contraction and enhance gastrointestinal motility, which may decrease the opportunities for ETEC colonization of the intestine.

Toll-like receptors (TLRs) play an important role in the regulation of innate immunity in animals. Numerous pathogenic molecules have been reported to be recognized by TLRs. For example, TLR4 proactively identifies the lipopolysaccharide (LPS) of ETEC, while TLR5 and TLR9 recognize flagellin and CpG-DNA, respectively (Cario, 2005; Lu et al., 2008; Kim et al., 2015). The activation of TLRs leads to the secretion of a large number of proinflammatory cytokines via the myeloid differentiation factor 88 (MyD88)/nuclear factor-kappa B (NF-κB) signaling pathway (Kawai and Akira, 2007). The present study showed that seven days after ETEC challenge, the relative mRNA expression of MyD88 in the jejunum was significantly upregulated in the piglets challenged with ETEC. Similarly, TLR5 in the ileum and TLR4, TLR5, TLR9, TRAF6, and NF-κB in the MLN were significantly upregulated. However, most of these genes (except TLR4 and TRAF6 in MLN) were not altered in the piglets given dietary supplementation of ATB or AN. To maintain the stability of the immune system, the MyD88/NF-κB signaling pathway is negatively regulated by TOLLIP and SIGIRR (Burns et al., 2000; Wald et al., 2003). In this study, the relative mRNA expression of SIGIRR in the jejunum, ileum, and MLN and the relative mRNA expression of TOLLIP in the ileum were upregulated to varying degrees with the administration of AN and ATB. Additionally, no significant difference in the MLN and TOLLIP mRNA expression was identified between the AN and ATB group. These results indicated that the AN supplements had functions similar to those antibiotics, which can inhibit the MyD88/NF-κB signaling pathway by activating its negative regulators. This has shown direct evidence that AN alleviates the inflammation induced by ETEC challenge in piglets.

Activated NF-κB regulates the expression of proinflammatory cytokines (Kawai and Akira, 2007). In the present study, we found that the mRNA expression of IL-1β and TNF-α in the jejunum, ileum and MLN was increased to varying degrees after ETEC challenge. The concentrations of IL-1β and TNF-α in the plasma also tended to increase. Elevated concentrations of IL-1β and TNF-α generate heat and lead to elevated rectal temperature (Yi et al., 2005; Tesch et al., 2018). This observation might partially explain the rapid increase in the intestinal temperature of piglets in response to intestinal infection in our study. Tight junctions (TJs) are the most important connections between cells, TJs only allow soluble, small molecule substances to pass through them, which hinders the passage of macromolecular substances and microorganisms (Lee, 2015), Excessive production of the proinflammatory cytokines IL-1β and TNF-α also break tight junction and increase cell bypass permeability in the gut (McKay and Baird, 1999; Ma et al., 2004; Al-Sadi et al., 2010). In addition, mucin protects the biological function of epithelial cells and participates in the processes of epithelial cell renewal and cell signaling activation, studies have found that downregulated mucin-1 can increase TNF-α expression (Guang et al., 2010). In our study, we observed the disruption of tight junctions and mucin secretion in response to ETEC infection. It is worth noting that AN is not the only essential oil that can regulate the inflammation of the intestine. In previous studies, thymol and oregano were also found to significantly alleviate the increase in IL-1β and TNF-α in the piglet jejunum mucosa caused by challenge with ETEC (Pu et al., 2018).

As we known, intestinal inflammation and gut microorganisms have relations, to investigate the effects of AN on the proliferation of intestinal microbiome in piglets, the microbes in the feces were analyzed by high-throughput 16S rDNA sequencing. In this study, the alpha diversity of the fecal microbiota in the AN group was significantly higher than that in the CON and ETEC group. Beta diversity showed that the microbiota from the ETEC group obviously tended to separate from that of both the ATB and AN group. Thus, the AN group had more similar microbial structures than the ATB group, which is different from the ETEC group. This evidence indicates that AN supplements have functions similar to those of antibiotics in modifying the structure of the intestinal flora. Specifically, we found the ATB group exhibited a significantly increased abundance of *Lactobacillus*, while the AN group exhibited a significantly increased abundance of *Selenomonas*, and both the ATB and AN group exhibited a significantly reduced abundance of unidentified_Ruminococcaceae. Under the normal condition, *Lactobacillus* can inhibit the TLR4 inflammatory signaling triggered by ETEC, which is conducive to the maturation of the intestinal mucosal immune system and triggers local immunomodulatory activity (Zhang et al., 2011; Finamore et al., 2014). *Selenomonas* can produce SCFAs which inhibit inflammation and enhance barrier function (Bladen et al., 1961; Rajilić-Stojanović et al., 2015). Recently study was found that Ruminococcaceae is a biomarker of microbes in oxidative damage and is highly abundant in many intestinal injury models (Zhou et al., 2018). Moreover, several studies have shown that Ruminococcaceae could be involved in recovery after ETEC infection (Salonen et al., 2010; Rajilić-Stojanović et al., 2015). These signs indicated that AN has a positive regulatory effect on intestinal microbiota of piglets infected with ETEC, but its mechanism may be different from the antibiotics. Overall, dietary supplementation with AN enriches the abundance of beneficial flora in the intestines of piglets, which enhances the intestinal functions of piglets and reduces the occurrence of inflammation.

## Conclusion

In summary, AN can attenuate enterotoxigenic *E. coli*-induced intestinal barrier disruption and intestinal inflammation via modification of TLR signaling and intestinal microbiota, then improving the growth performance of weaned piglets infected by ETEC. Meanwhile, AN is a promising alternative to antibiotics in animal husbandry.

## Data Availability Statement

The datasets presented in this study can be found in online repositories. The name of the repository and accession number can be found below: National Center for Biotechnology Information (NCBI) Sequence Read Archive (SRA), https://www.ncbi.nlm.nih.gov/sra, SRR13728343.

## Ethics Statement

The animal study was reviewed and approved by South China Agricultural University Animal Care and Use Committee.

## Author Contributions

QY was the principal investigator that designed the study, wrote the manuscript, carried out the animal trials, sample analysis, data collection, and statistical analysis. JL, YZ, and HQ carried out the animal trials and sample analysis. FC supervised the study. SZ revised the manuscript. WG designed and supervised the study and revised the manuscript. All authors read and approved the final manuscript.

## Conflict of Interest

The authors declare that the research was conducted in the absence of any commercial or financial relationships that could be construed as a potential conflict of interest.

## Supplementary Material

Supplementary Figure 1 The experimental procedure.

Supplementary Table 1 Composition and nutrient levels of basal diets (as fed-basis).

Supplementary Table 2 Primer sequences of target and reference genes.

Supplementary Table 3 The relative abundances of 8 phyla.

Supplementary Table 4 The relative abundances of 30 genera.

## Abbreviations

ADFI: average daily feed intake
ADG: average daily gain
AN: anethole
ATB: antibiotics
BW: body weight
CON: control
ETEC: enterotoxigenic *Escherichia coli*
F/G: feed conversion ratio
IL-1 β: interleukin-1 β
IL-6: interleukin-6
IL-10: interleukin-10
LPS: lipopolysaccharide
MLN: mesenteric lymph nodes
MyD88: myeloid differentiation factor 88
NF- κ B: nuclear factor kappa B
NMDS: Non-metric multi-dimensional scaling
OTU: operational taxonomic unit
PCoA: principal coordinate analysis
SEM: standard error of mean
SIGIRR: single immunoglobulin and toll-interleukin 1 receptor domain
TLR: toll like receptor
TNF- α: tumor necrosis factor- α
TOLLIP: toll interacting protein
TRAF6: tumor necrosis factor receptor-associated factor 6
VCR: villi height and crypt depth ratio
ZO-1: Zonula occludens protein-1.
