# Supplementary material for: Anethole Attenuates Enterotoxigenic Escherichia coli-Induced Intestinal Barrier Disruption and Intestinal Inflammation via Modification of TLR Signaling and Intestinal Microbiota
Source: Front Microbiol. 2021 Mar 25;12:647242. doi: 10.3389/fmicb.2021.647242 (PMC8027122; doi:10.3389/fmicb.2021.647242)
Supplement: Supplementary Figure 1 — The experimental procedure. [file Table_1.DOCX]

**Supplemental data**

Anethole attenuates enterotoxigenic *Escherichia coli-*induced intestinal barrier disruption and intestinal inflammation via modification of TLR signaling and intestinal microbiota

Qingyuan Yi§, Jiaxin Liu§, Yufeng Zhang§, Hanzhen Qiao¶, Fang Chen§‡，Shihai Zhang§‡*, and Wutai Guan§‡*

| **Table S1.** Composition and nutrient levels of basal diets (as fed-basis, %) | | | |
| --- | --- | --- | --- |
| ingredient | content | nutrient levels | content |
| Corn (7.8%, CP) | 44.00 | Digestible energy，MJ/kg | 14.58 |
| Wheat powder (15.3%, CP) | 10.00 | CP (%) | 19.28 |
| Soybean meal (43%, CP) | 16.00 | Crude fat (%) | 6.99 |
| Fish meal (62.5%, CP) | 8.50 | Crude fiber (%) | 2.41 |
| Whey (12%, CP) | 7.50 | Ash (%) | 5.74 |
| Glucose | 1.25 | Calcium (%) | 0.80 |
| Sucrose | 3.50 | Phosphorus (%) | 0.65 |
| Soybean oil | 4.50 | SID Lys (%) | 1.43 |
| CaHPO4 | 0.40 | SID Met+Cys (%) | 0.82 |
| Ca(HCOO)2 | 0.85 | SID Thr (%) | 0.87 |
| Salt | 0.30 | SID Trp (%) | 0.23 |
| ZnO (71.25%, Zn) | 0.20 | SID Leu (%) | 1.43 |
| Choline chloride (50%) | 0.10 | SID Ile (%) | 0.70 |
| L-Lysine HCl (98.5%) | 0.57 | SID Val (%) | 0.91 |
| DL-Methionine (99%) | 0.28 |  |  |
| L-Threonine (98.5%) | 0.22 |  |  |
| L-Tryptophan (98.5%) | 0.05 |  |  |
| L-Valine (98.5%) | 0.14 |  |  |
| Antioxidant | 0.04 |  |  |
| Mould inhibitor | 0.20 |  |  |
| Mycotoxin absorbent | 0.10 |  |  |
| Premix^1^ | 1.00 |  |  |
| Bran | 0.30 |  |  |
| Total | 100.00 |  |  |

CP, crude protein

^1^Supplied per kilogram of diet: 11375IU of vitamin A, 3500IU of vitamin D_3_, 37.55IU of vitamin E, 3.50mg of vitamin of K, 7.98mg of vitamin B_1_, 12.38mg of riboflavin, 10.68mg of vitamin B_6_, 44.23μg of vitamin B_12_, 27.46mg of pantothenic acid, 58.78mg of niacin; 2.10mg of folacin, 0.27mg of biotin, 1717.34mg of choline.

Supplied per kilogram of diet: 76.93mg of Cu, 196.83mg of Fe, 54.39mg of Mn, 1599.98mg of Zn, 0.40mg of Se, 0.69mg of I, 0.69mg of Co, 0.69mg of Cr.

**Table S2.** Primer sequences of target and reference genes

| Gene | Genbank  Accession Number | Primer Sequences（5’→3’） | Product  Length |
| --- | --- | --- | --- |
| *IL-1β* | NM_214055.1 | TCTGCCCTGTACCCCAACTG | 64 |
|  |  | CCAGGAAGACGGGCTTTTG |  |
| *IL-6* | NM_001252429.1 | TGGCTACTGCCTTCCCTACC | 132 |
|  |  | CAGAGATTTTGCCGAGGATG |  |
| *IL-10* | NM_214041.1 | TCAAACGAAGGACCAGAT | 328 |
|  |  | GAAGATGTCAAACTCACCC |  |
| *TNF-α* | NM_214022.1 | CATCGCCGTCTCCTACCA | 199 |
|  |  | CCCAGATTCAGCAAAGTCCA |  |
| *TLR4* | NM_001113039.1 | GCCATCGCTGCTAACATCATC | 108 |
|  |  | CTCATACTCAAAGATACACCATCGG |  |
| *TLR5* | NM_001348771.1 | GAGCATTGACAGGGAAGCCT | 87 |
|  |  | TCTGGGTGCAAGAATGCGAT |  |
| *TLR9* | XM_005669564.3 | GGGAGACCTCTATCTCCGCT | 119 |
|  |  | TCAGGCTTTTGGGGAGGTTG |  |
| *TRAF-6* | XM_013990069.2 | GCTGCATCTATGGCATTTGAAG | 71 |
|  |  | CCACAGATAACATTTGCCAAAGG |  |
| *SIGIRR* | NM_001315689.1 | ACCTGGGCTCCCGAAACTAC | 62 |
|  |  | GTCATCTTCTGACACCAGGCAAT |  |
| *TOLLIP* | NM_001315800.1 | CCCGCGCTGGAATAAGG | 74 |
|  |  | CATCAAAGATCTCCAGGTAGAAGGA |  |
| *MyD88* | NM_001099923.1 | TGGTAGTGGTTGTCTCTGATGA | 80 |
|  |  | TGGAGAGAGGCTGAGTGCAA |  |
| *NF-κB* | NM_001048232.1 | CTCGCACAAGGAGACATGAA | 147 |
|  |  | ACTCAGCCGGAAGGCATTAT |  |
| *Mucin-1* | NM_001204296.1 | ACACCCATGGGCGCTATGT | 68 |
|  |  | GCCTGCAGAAACCTGCTCAT |  |
| *Mucin-2* | XM_007465997.1 | CTGCTCCGGGTCCTGTGGGA | 100 |
|  |  | CCCGCTGGCTGGTGCGATAC |  |
| *Claudin-1* | NM_001244539 | TCTTAGTTGCCACAGCATGG | 106 |
|  |  | CCAGTGAAGAGAGCCTGACC |  |
| *Occludin* | NM_001163647 | TTCATTGCTGCATTGGTGAT | 113 |
|  |  | ACCATCACACCCAGGATAGC |  |
| *ZO-1* | XM_021098896.1 | GAGGATGGTCACACCGTGGT | 169 |
|  |  | GGAGGATGCTGTTGTCTCGG |  |
| *GAPDH* | NM_001206359 | ACTCACTCTTCCACTTTTGATGCT | 100 |
|  |  | TGTTGCTGTAGCCAAATTCA |  |

**Table S3.** The relative abundances of 8 phyla (%, > 1% in at least one sample) in the

fecal of weaned piglets

| Items | Treatments | | | | SEM | *P*-value |
| --- | --- | --- | --- | --- | --- | --- |
|  | CON | ETEC | ATB | AN |  |  |
| *Firmicutes* | 67.54 | 72.39 | 76.01 | 70.70 | 2.05 | 0.562 |
| *Bacteroidetes* | 19.86 | 18.64 | 18.29 | 21.35 | 1.66 | 0.928 |
| *Spirochaetes* | 5.79 | 2.58 | 0.88 | 1.34 | 0.78 | 0.093 |
| *Actinobacteria* | 2.36 | 3.38 | 1.45 | 2.41 | 0.38 | 0.390 |
| *Euryarchaeota* | 0.42 | 0.74 | 0.39 | 1.73 | 0.30 | 0.387 |
| *Proteobacteria* | 1.62 | 0.76 | 1.77 | 1.52 | 0.24 | 0.490 |
| *Tenericutes* | 1.05 | 1.14 | 0.85 | 0.61 | 0.19 | 0.794 |
| *Fibrobacteres* | 1.07 | 0.15 | 0.19 | 0.06 | 0.18 | 0.155 |

SEM, standard error of mean (n = 5).

Different letters mean statistically significant difference among the groups (*P* < 0.05).

**Table S4.** The relative abundances of 30 genera (%, > 1% in at least one sample) in the

fecal of weaned piglets

| Items | Treatments | | | | SEM | *P*-value | |
| --- | --- | --- | --- | --- | --- | --- | --- |
|  | CON | ETEC | ATB | AN |  |  |  |
| *unidentified_Clostridiales* | 12.92 | 7.53 | 17.32 | 15.13 | 2.29 | 0.504 |  |
| *Catenibacterium* | 5.86 | 5.56 | 1.16 | 2.04 | 1.44 | 0.589 |  |
| *Blautia* | 2.24 | 5.95 | 3.00 | 2.12 | 0.73 | 0.219 |  |
| *Lactobacillus* | 2.51^ab^ | 1.75^b^ | 8.71^a^ | 4.00^ab^ | 0.80 | 0.002 |  |
| *Terrisporobacter* | 2.89 | 3.83 | 2.88 | 2.86 | 0.63 | 0.944 |  |
| *Catenisphaera* | 1.70 | 3.96 | 0.67 | 0.73 | 0.57 | 0.141 |  |
| *unidentified_Ruminococcaceae* | 3.41^b^ | 5.42^a^ | 3.50^b^ | 2.79^b^ | 0.35 | 0.035 |  |
| *unidentified_Lachnospiraceae* | 3.21 | 3.32 | 2.47 | 2.22 | 0.31 | 0.547 |  |
| *Selenomonas* | 2.08^ab^ | 0.71^b^ | 2.83^ab^ | 4.02^a^ | 0.48 | 0.084 |  |
| *Subdoligranulum* | 1.52 | 3.23 | 1.95 | 1.59 | 0.32 | 0.212 |  |
| *unidentified_Spirochaetaceae* | 2.03 | 1.13 | 0.68 | 0.84 | 0.31 | 0.448 |  |
| *Megasphaera* | 1.71 | 0.40 | 1.28 | 2.54 | 0.35 | 0.174 |  |
| *Agathobacter* | 1.47 | 0.55 | 2.37 | 1.92 | 0.30 | 0.172 |  |
| *Methanosphaera* | 0.04 | 0.00 | 0.00 | 0.96 | 0.24 | 0.429 |  |
| *Faecalibacterium* | 0.75 | 1.25 | 1.80 | 1.28 | 0.20 | 0.347 |  |
| *Succinivibrio* | 0.91 | 0.53 | 0.99 | 0.27 | 0.20 | 0.575 |  |
| *Olsenella* | 0.91 | 0.72 | 0.17 | 0.49 | 0.18 | 0.537 |  |
| *Alloprevotella* | 0.87 | 0.18 | 0.45 | 1.21 | 0.19 | 0.212 |  |
| *Collinsella* | 0.89 | 1.81 | 0.61 | 1.27 | 0.20 | 0.166 |  |
| *unidentified_Prevotellaceae* | 1.14 | 1.27 | 1.51 | 1.32 | 0.19 | 0.927 |  |
| *Oribacterium* | 1.33 | 0.56 | 0.36 | 0.36 | 0.20 | 0.259 |  |
| *Fibrobacter* | 1.07 | 0.15 | 0.19 | 0.06 | 0.18 | 0.155 |  |
| *Oscillospira* | 0.12 | 0.11 | 0.59 | 1.06 | 0.15 | 0.060 |  |
| *Negativibacillus* | 0.29 | 1.23 | 0.44 | 0.58 | 0.14 | 0.078 |  |
| *Methanobrevibacter* | 0.37 | 0.74 | 0.39 | 0.77 | 0.16 | 0.766 |  |
| *Holdemanella* | 0.72 | 0.85 | 0.73 | 0.47 | 0.15 | 0.855 |  |
| *Parabacteroides* | 0.71 | 0.35 | 0.28 | 0.41 | 0.09 | 0.355 |  |
| *Turicibacter* | 0.00 | 0.04 | 0.18 | 0.32 | 0.08 | 0.533 |  |
| *Desulfovibrio* | 0.44 | 0.09 | 0.09 | 0.13 | 0.07 | 0.263 |  |
| *Peptoclostridium* | 0.00^b^ | 0.00^b^ | 0.44^ab^ | 0.56^a^ | 0.09 | 0.046 |  |

SEM, standard error of mean (n = 5).

Different letters mean statistically significant difference among the groups (*P* < 0.05).


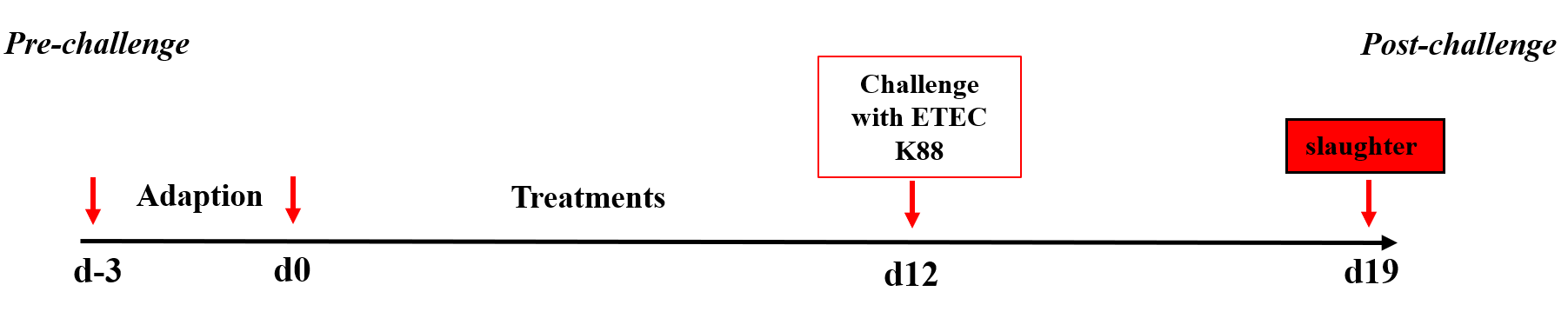


**Fig. S1.** The experimental procedure
